# Hypnoanalgesia and the study of pain experience: from Cajal to modern neuroscience

**DOI:** 10.3389/fpsyg.2014.01126

**Published:** 2014-09-30

**Authors:** Renzo C. Lanfranco, Andrés Canales-Johnson, David Huepe

**Affiliations:** ^1^Department of Psychiatry and Mental Health, Faculty of Medicine, Universidad de ChileSantiago, Chile; ^2^Laboratory of Cognitive and Social Neuroscience, UDP-INECO Foundation Core on Neuroscience, Universidad Diego PortalesSantiago, Chile; ^3^Medical Research Council, Cognition and Brain Sciences UnitCambridge, UK

**Keywords:** hypnosis, hypnotic suggestion, analgesia, Santiago Ramón y Cajal, cognitive neuroscience, consciousness, history

## Abstract

Santiago Ramón y Cajal (1852–1934) did not only contribute to neurobiology and neurohistology. At the end of the 19th century, he published one of the first clinical reports on the employment of hypnotic suggestion to induce analgesia (hypnoanalgesia) in order to relieve pain in childbirth. Today, the clinical application of hypnoanalgesia is considered an effective technique for the treatment of pain in medicine, dentistry, and psychology. However, the knowledge we have today on the neural and cognitive underpinnings of hypnotic suggestion has increased dramatically since Cajal’s times. Here we review the main contributions of Cajal to hypnoanalgesia and the current knowledge we have about hypnoanalgesia from neural and cognitive perspectives.

## INTRODUCTION

Hypnoanalgesia, i.e., the use of hypnotic suggestion to relieve pain, is widely accepted as an effective technique for the treatment of pain. However, the clinical use of hypnoanalgesia is not new. Throughout history, several cases of people using hypnosis to relieve pain have been documented. Some of the most remembered are Anton Mesmer (1734–1815; Mesmer, 1974; Radovancevic, 2009), John Elliotson (1791–1868; Elliotson, 1843), James Esdaile (1808–1859; Esdaile, 1852), and James Braid (1795–1860; Braid, 1850). However, one of the very first clinically detailed publications reporting the analgesic properties of hypnotic suggestion for pain relief was authored by Santiago Ramón y Cajal (1852–1934), the famous Spanish physician best known for his exceptional contributions to neurobiology and neurohistology. Here we review a less well-known side of Cajal: his particular way of employing hypnotic suggestion in order to induce analgesia. We also explore how the clinical use of hypnoanaglesia has evolved since then, and what modern neuroscience tells us about the neural and cognitive underpinnings of the hypnotic phenomena.

## BIOGRAPHICAL NOTE: SANTIAGO RAMÓN Y CAJAL

**FIGURE 1:**
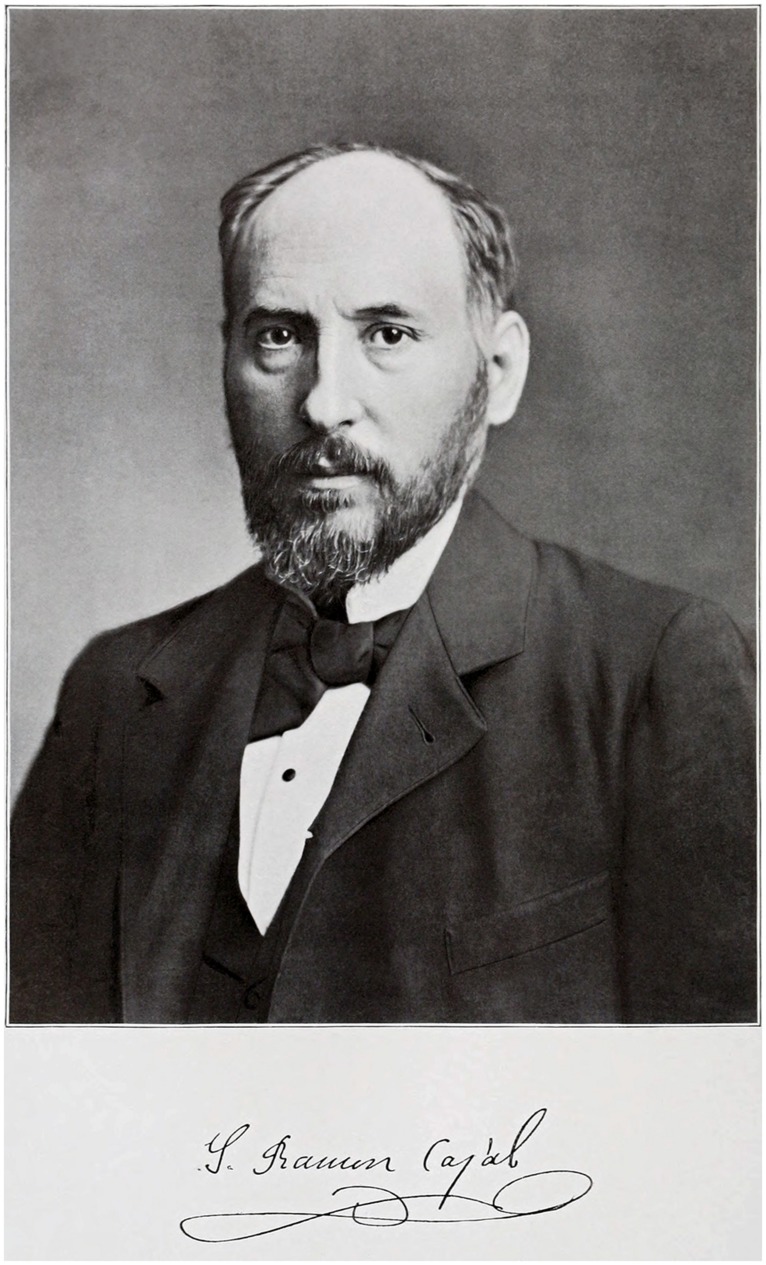
**Santiago Ramón y Cajal, 1899**.

Cajal was born on the 1st May 1852 in the town of Petilla de Aragón, Navarra, Spain. He lived in several towns during his childhood and later studied medicine in the city of Zaragoza. Unlike his academic performance during his schooldays, he excelled academically during his university studies, having been inspired to study medicine by his father, an anatomy professor at the University of Zaragoza. In 1875, Cajal started his doctoral studies in Zaragoza and began teaching histology in Madrid. Later on, Cajal was appointed professor of General and Descriptive Anatomy at the University of Valencia (1883), professor of Histology and Pathology in Barcelona and Madrid (1892), and director of the National Institute of Hygiene (1901; Ramón y Cajal, 1889) (see **Figure 1**).

Throughout his scientific career, Cajal was interested in a myriad of intellectual activities. He dedicated most of his time to research anatomy, physiology and morphology, especially of the nervous system and its connections. Based on his past findings, he proposed what later became known as the “neuron doctrine” – an idea that encompassed laws and theories about nerve impulses and the neurobiological organization of the brain, nowadays widely known. He published many articles on the topic and was awarded countless honorary doctorates and prizes, including the Nobel Prize for Physiology and Medicine in 1906, along with the Italian physician Camillo Golgi (González, 2006).

## CAJAL’S INTEREST IN HYPNOSIS

Cajal had many interests besides neurobiology. He was also passionate about photography, chess, and literature. He wrote both essays and novels, including a story about a hypnotherapist who attempted to create a utopia by using hypnosis (Stefanidou et al., 2007; Sala et al., 2008).

Moreover, Cajal had a fervent interest in psychological phenomena and higher cortical functions, particularly consciousness. He was fascinated by hypnosis for most of his life, keeping track of research and discoveries in the field, the major contributors being his contemporaries Charcot (of Salpêtrière school), and Liébeault and Bernheim (of Nancy school). But it was during his time at the University of Valencia (1884–1887) when Cajal’s interests in hypnosis really took off. Cajal organized, along with his wife and friends from the gatherings of the Agricultural Club, a “*Committee of Psychological Research*” that was held in Cajal’s own home. There, he carried out several hypnotic experiments with both healthy people and people with mental disorders, as well as with people who claimed to have mental powers, such as spiritual mediums (Ramón y Cajal et al., 1966; Ramón y Cajal, 2002; Stefanidou et al., 2007).

Cajal was interested in several hypnotic phenomena, including cataplexy; analgesia; visual, auditory and tactile hallucinations; and amnesia. He proved to be extremely skillful in inducing hypnosis and guiding imagery to his patients, thus becoming rather popular amongst patients who were suffering from hysteria and neurosis. Amongst his major accomplishments were: “*the total transformation of the patient’s emotional state, restoration of appetite in hysterical-epileptic patients with loss of appetite, sudden cessation of hysterical attacks with loss of consciousness, radical forgetfulness of painful and tormenting events, and the complete abolition of delivery pain in normal women*” (Ramón y Cajal et al., 1966).

Years later, Cajal closed his clinic due to taking a new academic position in Barcelona (Ramón y Cajal, 2002; Stefanidou et al., 2007).

## CAJAL AND THE USE OF HYPNOANALGESIA IN CHILDBIRTH

Even though the first reports of using hypnoanalgesia may be attributed to James Esdaile – an English surgeon who practiced in India (Esdaile and Esdaile, 1846; Gauld, 1992), the first case study on the use of hypnoanalgesia in labor and delivery was published by Ramón y Cajal (1889). It consisted of a clinical case study of his own wife, Silveria Fañanás, who had been preparing to give birth to their sixth child, Pilar, in Barcelona. Wishing to avoid seeing his wife suffer the pain like in her previous childbirths, Cajal proposed that she used hypnosis as a pain relief method, which she accepted. Thus, both their daughter Pilar, and their last child Luis, were born whilst their mother was under hypnosis (Ramón y Cajal, 2002).

Ramón y Cajal’s (1889) article was published in the *Catalan Medical Gazette* under the title: “*Pain of childbirth considerably attenuated by hypnotic suggestion*.” Unfortunately, the publication went largely unnoticed. It received only a brief and anonymous comment in the British Medical Journal (1889). Much later, Stefanidou et al. (2007) presented an English translation that finally allowed its dissemination:

Cajal describes a woman who had been prepared for hypnoanalgesia 10 days prior to her childbirth. She was able to reach an alleged state of somnambulism, characterized by anesthesia, catalepsy and subsequent amnesia. Cajal reports to have had carried out a pre-tested method in order to attenuate her pain. Furthermore, Cajal argues the little resistance that the patient exhibited to hypnotic suggestions as believing the technique to be harmless was further attenuated by her fear of childbirth pain. According to Cajal, the hypnotic suggestions employed revolved around the idea of how quickly the delivery was going to be, and that even if contractions were strong and constant, the pain would remain minimal and utterly tolerable: “*We told her that she would be conscious of the stronger pain produced both by the cervical dilation, and during the fetus expulsion; but the severity of that pain would be difficult to distinguish from the weak pain known as preparative or ‘moscas’ [pain of the very first contractions]*” (Ramón y Cajal, 1889; Stefanidou et al., 2007).

According to Cajal’s report, the patient’s only perceived discomfort during childbirth that never turned into pain. In fact, it seemed to Cajal that the discomfort that she felt was due to respiratory distress and fast heart rate related to the intense physical work involved in childbirth, rather than pain (Ramón y Cajal, 1889). Thus, to the patient’s great surprise, her cervix was fully dilated and the birth was completed in <30 min (Stefanidou et al., 2007). Moreover, the patient’s recovery was rather fast as well; in 5 days she was already back on her feet and returned to her daily activities.

Ramón y Cajal’s (1889) final remarks highlighted how useful hypnosis could be in attenuating childbirth pain without causing any of the organic alterations seen as side effects of chloroform-induced sleep.

## CAJAL’S FINAL YEARS USING HYPNOSIS

During his last years, Cajal abandoned his neurobiological research in order to focus on the study of dreams and spiritualism; he even hired an alleged medium from Zaragoza to carry out experiments. However, later on he realized that the medium was a fraud. Notwithstanding the foregoing, Cajal maintained the belief that hypnosis may connect mind and matter, thus forming a method capable of producing profound neurobiological changes (Gamundí et al., 1995). As Ramón y Cajal (1947) himself stated: “*I do not consider [to be] … unrealistic … the achievement of a mental orthopedics capable of correcting the functional aberrations of the brain; on the contrary, I judge it possible that, dispelling certain prejudices, physiology, assisted by methods of psychophysical hypnosis and scientific pedagogy, could eliminate antisocial impulses or reduce them to a negligible minimum*.”

Ramón y Cajal (2002) also included his experiences employing hypnoanalgesia in a collection of his works in 1924, in a book entitled “Ensayos sobre el hipnotismo, el espiritualismo y la metafísica” [“Essays on hypnotism, spiritualism and metaphysics”], in which he gathered hundreds of analyses of his own dreams and others’. Cajal finished the manuscript months before his death. Unfortunately, during the Spanish Civil War (1936), the Alfonso XIII Institute of Hygiene in Madrid, where the manuscript was kept, was severely damaged during a bombing and the document was lost forever (Ramón y Cajal, 1889; Sala et al., 2008).

## CURRENT RESEARCH IN HYPNOTIC MODULATION OF PAIN EXPERIENCE

Since Cajal’s early contributions to the clinical practice of hypnoanalgesia, there has been a gradual increase in scientific interest in the efficacy of its clinical application as compared to other analgesic strategies. Another line of research has focused on its neural and cognitive underpinnings, posing the question of what hypnotic suggestion does to brain function.

Hypnotically suggested algesia has been employed to identify the brain mechanisms that are directly associated to the emotional component of pain experience. Rainville et al. (1997), for instance, used hypnotic suggestion to dissociate the affective from the sensory aspects of pain. In a positron emission tomography (PET) study, they demonstrated that the anterior cingulate cortex (ACC) showed to be deeply involved in such functional dissociation. Furthermore, Derbyshire et al. (2004) used hypnotic suggestion to induce pain in the absence of a noxious stimulus. Using functional magnetic resonance imaging (fMRI), they showed that the pain matrix activity (i.e., brain regions recruited in pain experience, such as ACC, insular cortex, secondary somatosensory cortex, dorsolateral prefrontal cortex) was increased by hypnotic suggestions of pain in a greater way than during imagining of pain. According to the results, hypnotic suggestion to induce pain would decrease the activation in the perigenual ACC, a region that has been related to internal monitoring of sensory information (Porro et al., 2002). Thereafter, other investigations were carried out in order to elucidate the brain mechanisms underlying the hypnotic modulation of pain experience. For example, Raij et al. (2009), showed, using fMRI, that the right dorsolateral prefrontal cortex (dlPFC), which is involved in modulating the brain’s pain matrix, has an increase in activity during hypnotic suggestion. Given that dlPFC activation strength also predicts the effectiveness of placebo analgesia (Wager et al., 2004) and has been widely reported to be related to cognitive control as well (MacDonald et al., 2000; Hutcherson et al., 2012; Lesh et al., 2013), it seems feasible it may have an important role in modulating other regions related to pain experience, such as secondary somatosensory cortex. However, further research is required in this regard.

**FIGURE 2:**
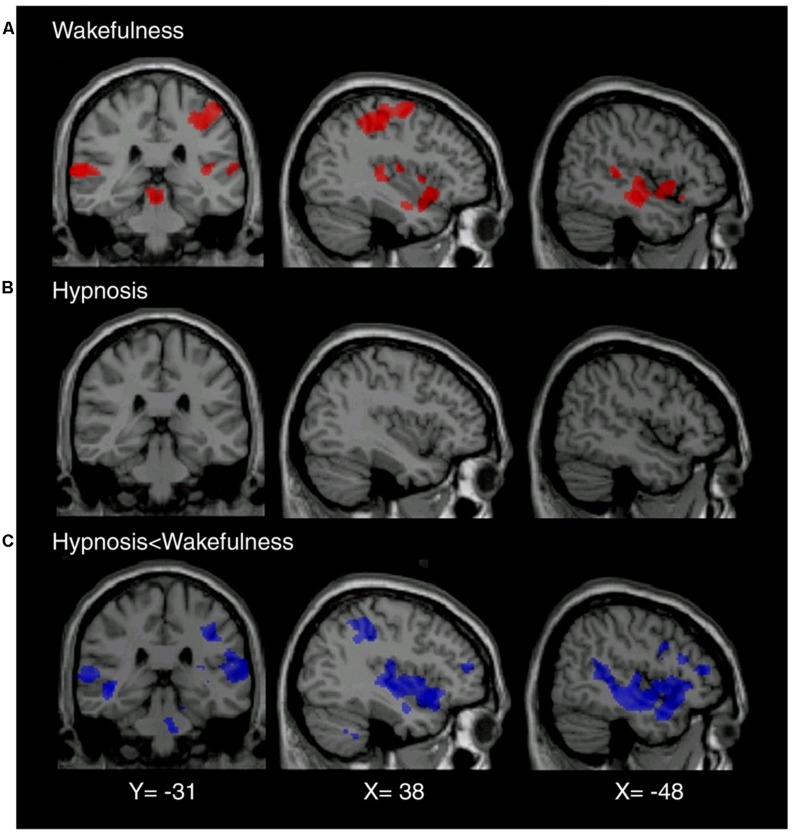
**(A)** Brain regions showing significant (*p* < 0.05) activation during noxious stimulation (≥450 mJ) in normal wakefulness (mean laser intensity 534 ± 8 mJ). **(B)** In the hypnotic state, intensity-matched sensory stimuli failed to elicit any cerebral activation. **(C)** Brain regions showing significant differences with activation induced by identical stimuli (mean laser intensity 532 ± 14 mJ) in hypnotic state. Reproduced with permission of Elsevier, Inc.

Similarly, hypnotically suggested analgesia has also been employed in studying neural mechanisms underlying pain experience and relief. Faymonville et al. (2000), using PET, showed that hypnoanalgesia decreases both pain sensation and unpleasantness of noxious stimuli by activating ACC and right-sided extrastriate (which have been related to stress symptoms) and decreases thalamic nuclei activity. ACC has been associated to social pain (i.e., unpleasant experience related to actual or potential damage to one’s sense of social connection or social value) and to the affective component of pain (Fuchs et al., 2014; Rotge et al., 2014). Other studies have presented supplementary results upon the subjective experience of pain (Faymonville et al., 2000; Raij et al., 2005). For instance, using a thulium-YAG laser to induce pain, Vanhaudenhuyse et al. (2009) explored activation within the pain matrix when comparing painful and non-painful stimulation. As expected, activity within the pain matrix was significantly decreased during hypnoanalgesia (see **Figure 2**).

In addition, studies using electroencephalography (EEG) have also helped to unveil intrinsic and neurological aspects of pain experience and how it is modulated by hypnoanalgesia. For instance, it has been shown that late event-related potentials (ERPs) waveforms make a difference when comparing high and low hypnotically susceptible individuals during hypnoanalgesia (Ray et al., 2002). These results might signify higher top-down modulation in highly hypnotizable people when compared to lower hypnotizable people. Moreover, significant reductions in phase-ordered gamma patterns have been reported in medium to high hypnotizable individuals while performing a pain task during hypnoanalgesia. The pattern was predictive of subjective pain ratings (De Pascalis et al., 2004b). Gamma activity has been previously associated to early processing of stimulus information (Basar et al., 1987), and integration of sensory feature binding (Fell et al., 2003). There is evidence that has linked large-scale gamma-band phase synchronization to attention focusing (Doesburg et al., 2008), awareness (Rodriguez et al., 1999; Melloni et al., 2007), and cognitive control in meditation (Lutz et al., 2004, 2008). Reductions in phase-ordered gamma patterns might be taken as a marker of loss of cognitive control and metacognition, which would fit the cognitive state of someone successfully following hypnotic suggestions.

In another experiment, it was shown that hypnoanalgesia in high hypnotizable individuals elicited higher central peaks of the ERP P300, which was interpreted as a signal of an altered brain functioning. Such a conclusion would support the idea of a dissociated control, where hypnoanalgesic responses occurred with involuntariness (De Pascalis et al., 2004a). Recently, Del Percio et al. (2013) reported that some EEG features were related to hypnotizability rather than hypnotic suggestion. Furthermore, Valentini et al. (2013), recently demonstrated that hypnotic suggestion modulates both sensory and affective dimensions of the subjective experience of pain, especially in high hypnotizable participants. Hypnotic suggestions did not significantly affect early stages of sensory processing. However, late ERP components such as P2a and P2b showed increase and decrease wave amplitudes, respectively, and an increase in gamma band power, during unpleasantness manipulation.

The theories that have addressed the phenomenon of hypnotic suggestion could be classified into two groups: the “state” and the “non-state” theories. The former assume that a distinguishable neurobiological state is needed in order for hypnotic suggestions to yield its effects on cognition and consciousness. The latter postulate that hypnotic suggestions do not need a special neurobiological state to modulate cognition and consciousness. Even though experimental research involving hypnoanalgesia has certainly contributed to both clinical practice and neurobiological knowledge, we still lack of a theory that satisfactory explains the nature of hypnotic suggestions. For example, some studies show that hypnosis (as a state of consciousness) modulates specific brain regions (Rainville et al., 2002) and presents very specific eye movement patterns that may not be achieved during wakefulness (Kallio et al., 2011). Hypnotic suggestion has been shown to dissociate systems in charge of cognitive control and attentional conflict monitoring (Egner et al., 2005), while neutral hypnosis (i.e., the induction of hypnosis without further suggestions) has shown to decrease brain activity in the anterior portion of the default-mode network in high suggestible participants, but not in low suggestible participants (McGeown et al., 2009). In fact, neutral hypnosis has also shown to induce changes in several cortical regions and its activity patterns, including changes in functional connectivity (Fingelkurts et al., 2007). These studies, amongst many others (Kosslyn et al., 2000; Kallio et al., 2001; McGeown et al., 2012), suggest that even though our understanding on hypnotic suggestion has dramatically improved over the past decades, we are still unable to fully explain neither the hypnotic phenomena nor how hypnotic suggestions alter brain functions. Future integrative theories are required in this sense.

## DISCUSSION

Even though Cajal was not the first one to use hypnotic suggestion in order to relieve pain, to our knowledge he was the first one to publish a case report with such detailed clinical proceeding. Furthermore, it is notable that despite not proposing important theories for the understanding of hypnotic suggestion, Cajal’s procedure resembles very much the ones that clinicians use nowadays when treating pain in labor and delivery (VandeVusse et al., 2007; Landolt and Milling, 2011). Cajal prepared the patient weeks before the childbirth in order to build (or in his case, strengthen) the rapport. The suggestions he employed aimed to relieve pain, decrease emotional stress, and induce relaxation, whilst also assuring that the patient maintained her motor control to be able to push. In this sense, his publication may be taken as probably the first modern clinical intervention featuring hypnoanalgesia during childbirth.

According to his biographers, Cajal wrote several articles on hypnosis, spirituality and dreams, but he never published them. However, he was always up to date with the work of Charcot, Liébeault, Bernheim, and Freud (Ramón y Cajal et al., 1966). Sadly, his contributions to the study of hypnoanalgesia were completely forgotten, even by the Spanish obstetricians of the time.

Today, many core questions regarding the nature of hypnosis and hypnotic suggestion remain unanswered. Future research may focus on unveiling the neural and cognitive mechanisms underlying hypnotizability in order to create a better understanding of what is required for hypnoanalgesia to work. If hypnotic induction is not required for hypnotic suggestion to induce analgesia, then simpler techniques for the non-pharmacological treatment of pain might be possible in the future. However, there are terminological issues in this regard. Many studies do not distinguish between hypnotic suggestion, hypnosis as a specific state of consciousness, and hypnotic induction. This is an important limitation that readers should take into account when trying to understand the nature of the hypnotic phenomena, which also affects our ways to theorize about hypnosis and hypnotic suggestion. As Lynn and Rhue (1991) argued, hypnotic inductions are hypnotic suggestions, hence unless we are able to induce the so-called hypnotic state without using verbal suggestions, it becomes very difficult to defend the idea that under hypnotic suggestions there is a hypnotic state. Cajal did not clearly distinguish between hypnosis and hypnotic suggestion, although his publications suggest he believed in an altered state of consciousness working underneath suggestions (Ramón y Cajal, 1889; Ramón y Cajal et al., 1966).

Hypnoanalgesia has proved to be very effective in the treatment of pain, which includes chronic oncological pain (Cassileth and Keefe, 2010; Sohl et al., 2010), HIV neuropathic pain (Dorfman et al., 2013), pain during extraction of molars (Abdeshahi et al., 2013), pain associated to physical trauma (Patterson et al., 2010), pain in surgical procedures (Facco et al., 2013), pain associated to temporomandibular joint disorder (Abrahamsen et al., 2010), phantom limb (Mack et al., 2013), fibromyalgia (Derbyshire et al., 2009), pain in amyotrophic lateral sclerosis (Palmieri et al., 2012), acute pain in children (Yaster, 2010), lumbago (Tan et al., 2010, 2014), and pain in childbirth (Oster, 1994; VandeVusse et al., 2007; Abbasi et al., 2009), amongst others. This fact fits what Cajal suggested more than a century ago. It is motivating that the father of the “neuron doctrine” considered hypnotic suggestion a useful method for the treatment of pain. It would have probably pleased him to learn about the progress in understanding the neural substrates of hypnosis and hypnoanalgesia that follows from the work that won him the Nobel Prize.

## Conflict of Interest Statement

The authors declare that the research was conducted in the absence of any commercial or financial relationships that could be construed as a potential conflict of interest.
